# Effect of prehabilitation programmes on functional capacity in patients awaiting oncological resections: a systematic review and meta-analysis of randomised controlled trials

**DOI:** 10.1007/s00520-024-08875-8

**Published:** 2024-09-17

**Authors:** Roberto Laza-Cagigas, Eneko Larumbe-Zabala, Tara Rampal, Marcos Seijo, Fernando Naclerio

**Affiliations:** 1 Institute for Lifecourse Development, Centre for Exercise Activity and Rehabilitation, School of Human Science, University of Greenwich, Sparrows Farm (Office SF112B), Sparrows Lane, Avery Hill Campus, Eltham, SE9 2TB England, UK; 2 Department of Public Health, Fundación Canaria Instituto de Investigación Sanitaria de Canarias, Las Palmas de Gran Canaria, Spain; 3 QuestPrehab, London, UK

**Keywords:** Cancer, Surgery, Exercise

## Abstract

**Purpose:**

To investigate the effects of prehabilitation on the perioperative functional capacity of patients awaiting oncological resections.

**Methods:**

A systematic review and meta-analysis were performed in accordance with the Preferred Reporting Items for Systematic Reviews and Meta-Analysis (PRISMA) checklist and within the databases Cochrane Library, EBSCOhost, Google Scholar, MEDLINE PubMed, and Web of Science. The eligibility criteria were set to include peer-reviewed randomised control trials including only adult (≥ 18 years old) patients undergoing any type of prehabilitation (PREHAB) prior to any type of oncological resection. The studies had to feature at least one control group undergoing standard care (SC) and had to assess functional capacity by means of a 6-min walk distance (6MWD) or peak oxygen uptake (VO_2Peak_) at different stages pre- and post- operatively.

**Results:**

Twenty-seven randomised controlled trials involving 1994 patients were included. After processing the data, the number of patients was 1889. Studies featured different cancer specialties: lung (11), colorectal (5), urological (4), abdominal (3), esophagogastric (2), liver (1), and gastrointestinal (1). Overall, PREHAB enhanced both 6MWD (*g* = 0.273, 95% CI 0.174 to 0.371, *Z* = 5.406, *p* < 0.001) and VO_2Peak_ (*g* = 0.615, 95% CI 0.243 to 0.987, *Z* = 3.240, *p* = 0.001) compared with SC. The 6MWD subgroup analysis revealed a small mean effect size favouring both unimodal and multimodal PREHAB interventions.

**Conclusion:**

These findings support that prehabilitation, whether implemented as unimodal or multimodal format, elicits small preoperative improvements in functional capacity in patients awaiting oncological resections.

PROSPERO registration number CRD42023428676.

**Supplementary Information:**

The online version contains supplementary material available at 10.1007/s00520-024-08875-8.

## Introduction

The term prehabilitation (PREHAB) refers to the process of optimising patient health prior to surgery with the intention of improving postoperative outcomes [1]. Recently, some researchers and healthcare professionals have advocated for the implementation of consensuses nationally [2] and internationally [3] to determine the characteristics that preoperative PREHAB programmes should comprise. In this respect, in the UK, the incorporation of PREHAB into the pathway of patients expecting oncological resections has gained popularity and is currently being recommended as an effective approach to palliate the side-effects of non-surgical cancer treatment [2].

Prehabilitation programmes can comprise one component (e.g., physical exercise) or more than one (e.g., nutritional advice and psychological support), being referred to as unimodal and multimodal PREHAB, respectively. Furthermore, prehabilitation can be presented in various formats: in person and digitally; one-to-one and in groups; supervised and non-supervised; in the community, the hospital, and at home; and as a combination of all the above. This implies that although PREHAB might be the most prevalent term at the moment, any intervention conducted before surgery with the intention of improving patients’ overall health in preparation for surgery would fit the definition even when the term “prehabilitation” is not explicitly used (e.g., preoperative rehabilitation, nutritional therapy). The recent proliferation of publications covering PREHAB helps to obtain a deeper understanding of its potential benefits. In fact, recent studies have suggested that PREHAB may improve patients’ preoperative fitness [4, 5], and clinical outcomes [6, 7], which may in turn translate into reduced hospital costs [8, 9].

Previous randomised controlled trials and systematic reviews have investigated the effects PREHAB may elicit on, amongst other aspects, postoperative outcomes (e.g., mortality) and functional capacity [10–20]. Functional capacity reflects the ability to perform activities of daily living that require sustained aerobic metabolism [21], and has been commonly assessed by means of the maximum distance covered in 6 min (6MWD), or the maximum or peak oxygen uptake (i.e., VO_2max_ and VO_2Peak_, respectively) attained during a cardiopulmonary exercise test. It has been shown that oxygen demands are increased during the postoperative period and higher functional capacity can improve postoperative outcomes [22]. Therefore, if preoperative PREHAB programmes improve patients’ functional capacity prior to oncological resections, this intervention could also improve postoperative outcomes. A systematic review with broad eligibility criteria is needed to ascertain whether PREHAB improves preoperative functional capacity in patients diagnosed with cancer and expecting surgery as part of their cancer treatment.

The aim of this systematic review and meta-analysis was to summarise the effect that prehabilitation can elicit on functional capacity in patients awaiting oncological resections.

## Methods

This review was conducted in accordance with the Preferred Reporting Items for Systematic reviews and Meta-Analysis (PRISMA) checklist [23] and registered with the International Prospective Register of Systematic Reviews, PROSPERO (CRD42023428676).

### Search strategy

The procedures for the current systematic review and meta-analysis included: identification, screening, eligibility, and inclusion/exclusion of studies. A systematic search of the literature with no lower date limit was conducted by two reviewers (RLC and FN) within the following databases: Cochrane Library, EBSCOhost, Google Scholar, MEDLINE PubMed, and Web of Science, through January 2023 to May 2023. Articles had to be written in either English or Spanish.

Applying our search criteria, we identified all randomised controlled trials (RCT) addressing PREHAB in patients undergoing surgery as treatment for cancer. Our search results were supplemented by a manual search of studies included in previously published systematic reviews and meta-analysis [10–20] to ensure that all eligible studies had been included. Resources, without any assigned DOI, commentaries, reviews, or duplicate publications from the same study, were not included. The reference lists of the retrieved studies were hand-searched to identify potentially eligible studies not captured by the electronic searches. Two reviewers (RLC and FN) independently screened the title, abstract, and reference list of each study to locate potentially relevant studies. Any discrepancies between the two authors were resolved through consensus or by the opinion of a third author (ELZ).

Combinations of the following keywords were used as search terms: “(operation OR operative OR surgical OR surgery) AND (cancer OR oncological OR oncology) AND (prehabilitation OR physical activity OR exercise OR nutrition OR oral supplements OR supplementation OR oral nutritional supplements)”. After each search, duplicated records were removed.

### Eligibility criteria

We used the Population, Intervention, Comparison, and Outcomes (PICO) framework to determine the eligibility of the studies as follows:

#### Population

We considered studies on adults (age ≥ 18 years) of any sex, diagnosed with any cancer, and expecting surgery as curative treatment, with or without neoadjuvant therapy, independently of whether they smoked or drank alcohol.

#### Intervention

Eligible studies could feature PREHAB programmes with the following characteristics: unimodal (e.g., nutrition or physical exercise or anxiety coping strategies) and/or multimodal; supervised and/or non-supervised; face-to-face and/or virtual; hospital-, community-, and/or home-based; delivered to groups of patients and/or individually. The length of the PREHAB programme had to be at least 1 week.

#### Comparator

We included published, peer reviewed RCT with two or more arms, with at least one arm following a PREHAB programme (intervention group) and another group following the usual or standard care (control group).

#### Outcomes

Studies were considered for inclusion if they reported measurements of functional capacity (i.e., 6MWD and/or VO_2Peak_) at different time points during the study.

Studies including patients with and without cancer were included only if they met the rest of the eligibility criteria and data from only those patients diagnosed with cancer were successfully retrieved after contacting the authors.

### Study records

#### Data management and selection

Potentially relevant articles were selected by (i) screening the titles; (ii) screening the abstracts; and (iii) if abstracts did not provide sufficient data, the entire article was retrieved and screened to determine whether it met the inclusion criteria. When data were not presented in the desired format or accurately (i.e., as figures or graphs), we contacted the corresponding authors to request the data. If no answer was obtained, available figures/graphs were analysed using the PDF Adobe Acrobat Pro software measuring tool and means and standard deviations were estimated from medians and interquartile ranges using methods described elsewhere [24]. Where studies matched the selection criteria but included both patients with and without cancer, we contacted the authors to retrieve the data pertaining to only patients with cancer. Thereafter, RLC and FN decided whether the selected articles matched and fitted the purpose of the systematic review.

#### Data collection process and coding

Data were independently extracted by two authors (RLC and FN) using a standardised data extraction sheet, and any disputes were discussed and settled by a third author (ELZ). The following qualitative and quantitative information was extracted from each included study: (1) authors; (2) publication year; (3) cancer speciality (e.g., prostate, lung, colorectal, breast, etc.); (4) baseline population characteristics; (5) characteristics of the PREHAB intervention, including its duration and components; (6) control procedures; (7) blinding; (8) sample size per group; (9) group means and standard deviations (SD) for functional capacity outcomes at all the available time points. Mean and SD were requested from the corresponding authors when only other statistics were reported (e.g., median, and interquartile range).

### Risk of bias in individual studies

Methodological information regarding the potential impact of bias was critically examined. For each study, seven domains from the Cochrane collaboration tool for assessing the risk of bias [25] were scored with high, low, or unclear risk for bias: sequence generation, allocation concealment, blinding of participants and personnel, blinding of outcome assessment, incomplete outcome data, selective outcome reporting, and similarity in baseline characteristics. These domains aim to assess the level of risk regarding different sources of bias, respectively: selection bias, allocation bias, performance bias, detection bias, attrition bias, reporting bias, and other biases. Two reviewers (FN and RLC) performed the quality assessment independently, and their findings were compared and discussed until consensus was achieved. Each domain was scored as − 1 for high risk, 0 for unclear risk, and 1 for low risk. Scores were then summed with a possible range of scores from − 7 to 7.

### Data analysis

The Comprehensive Meta-Analysis Software, version 4.0.000 (Biostat Inc., Englewood, New York, USA), was used to perform the meta-analysis, the sensitivity analyses, and the publication bias analysis, and to generate forest and funnel plots. The random-effects model was selected based on the assumption of variability in the true effects between studies. Five or more studies were required to generate weighted group mean differences, 95% confidence intervals (CIs), *p*-values and prediction intervals, for the effect size (Hedges’ *g*). From the collected data, we used the means, standard deviations (SD), and sample sizes, available at different points in time (e.g., baseline, preoperative, postoperative) for both the PREHAB and the standard care (SC) groups. The primary meta-analysis compared the effects of any PREHAB intervention versus SC in 6MWD and VO_2Peak_ measured pre- and post-operatively. When possible, we conducted a subgroup analysis to evaluate the effect of PREHAB programmes whether they had been applied using a unimodal, or multimodal format. All primary and secondary effect sizes were interpreted using Cohen’s convention for small (0.2), medium (0.5), and large (0.8) effects [26, 27].

When a quantitative analysis was not possible, a summary of the critical facts and results of the observed outcomes was reported. If sensitivity analyses were significantly high, the data were not meta-analysed and only the individual results were reported instead. Additionally, the presence of studies with inflated standardised residual values (> 1.96 or <  − 1.96) was examined to consider them as outliers. Funnel plots of effect size (horizontal-axis) by the standard error (vertical-axis), and the “trim and fill” procedure for the random effects were used to assess publication bias.

## Results

### Study selection

**Fig. 1:**
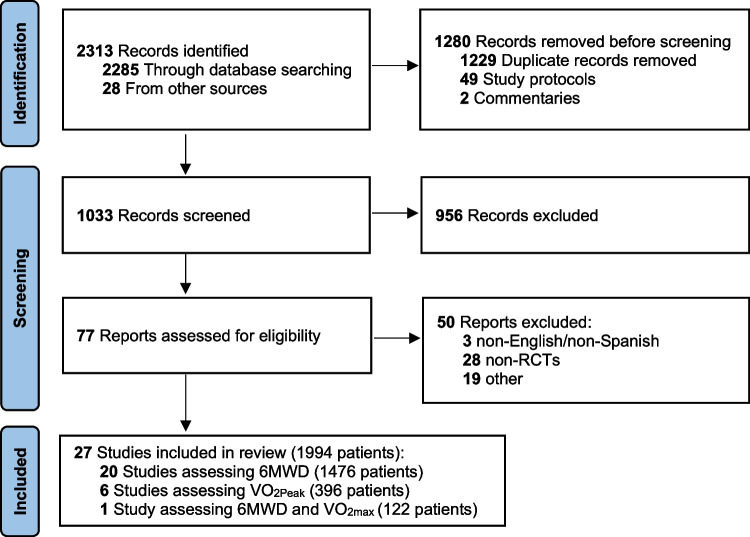
Preferred Reporting Items for Systematic Reviews and Meta-Analyses flow diagram for study selection

The search strategy is described in Fig. 1. The preliminary search identified 2313 relevant references. After examining all the retrieved records and deleting duplicates and other non-relevant records, 1033 publications were screened. Of those, 956 were excluded based on the title or abstract review, and the 77 records left were assessed for eligibility. After this examination, 50 studies were excluded resulting in a total of 27 studies [4–7, 28–50] to be included in the meta-analysis.

### Characteristics of the included studies

**Fig. 2:**
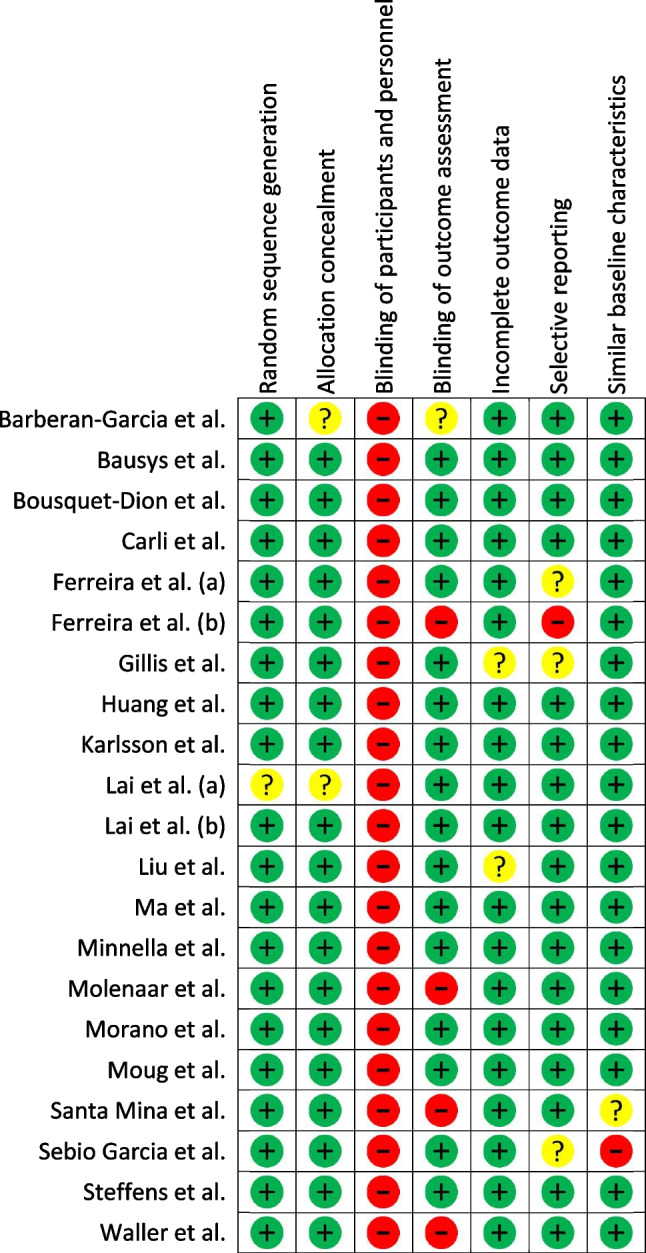
6-min walk distance risk of bias summary based on the Cochrane collaboration tool

**Fig. 3:**
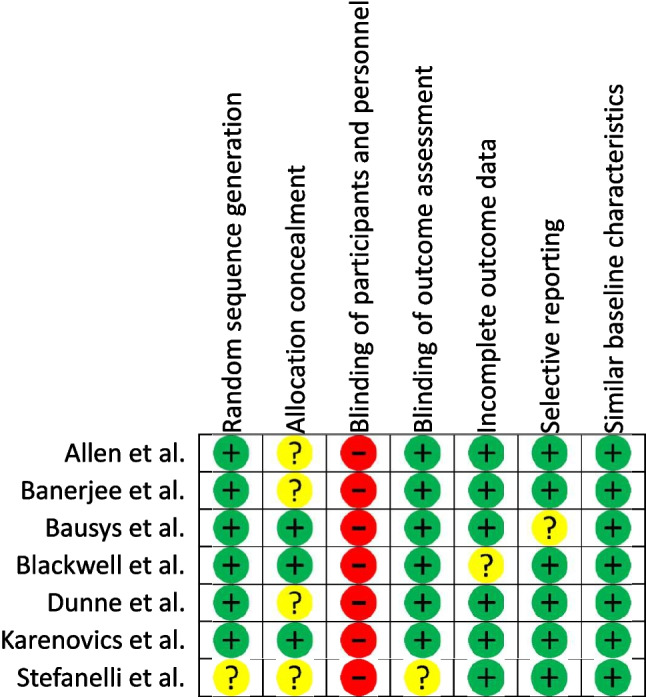
Peak oxygen uptake (VO_2Peak_) risk of bias summary based on the Cochrane collaboration tool

The overall quality of the included studies was high, with a low risk of bias, scoring from 1 to 5 points in the Cochrane collaboration tool [51]. The risk of bias for the 6MWD and the VO_2Peak_ is summarised in Fig. 2 and Fig. 3, respectively_._ The main characteristics of the studies are summarised in Table 1.

**Table 1:** Summary of the randomised controlled trials included in the meta-analysis

Study	Design	Participants	Length	Intervention	Measurements	Outcomes
Allen et al. [40]	Two parallel arms PREHAB (*n* = 26), SC (*n* = 28)	Esophagogastric; ≥ 18 y/o *n* = 54	15 Weeks	Exercise: aerobic, resistance, and flexibility training Nutritional: frequent, tailored, dietetic input Psychological: medical coaching with psychotherapist	VO_2Peak_	↑
Banerjee et al. [41]	Two parallel arm, feasibility, single-centre PREHAB (*n* = 30), SC (*n* = 30)	Bladder *n* = 60	mean SD 32 (6.5) days	Exercise: vigorous intensity interval aerobic training	VO_2Peak_	→
Barberan-Garcia et al. [48]	Blind, parallel arms PREHAB (*n* = 62), SC (*n* = 63)	Abdominal > 70 y/o *n* = 125	6 weeks	Exercise intervention: aerobic and resistance training Psychological: motivational interview	6MWD	?
Bausys et al. [49]	Two parallel arm, open-label, two centres PREHAB (*n* = 61), SC (*n* = 61)	Gastrointestinal ≥ 18 y/o *n* = 122	mean (SD) 92 (33) days	Exercise: aerobic, respiratory, resistance, and flexibility training Nutritional: nutritional counselling, ONS Psychological: oncopsychologist, relaxations techniques to do at home	6MWD VO_2max_	↑
Blackwell et al. [28]	Two parallel arms PREHAB (*n* = 19), SC (*n* = 21)	Urological *n* = 40	median [IQR] 30 [26–30] days	Exercise: supervised HIIT training	VO_2Peak_	↑
Bousquet-Dion et al. [29]	Two parallel-arm PREHAB + REHAB (*n* = 37), REHAB (*n* = 26)	Colorectal Adults *n* = 63	PREHAB: 4 weeks REHAB: 8 weeks	Exercise: aerobic and resistance training Nutritional: WPS, nutritional counselling Psychological: psychology-trained staff, exercises to do at home	6MWD	→
Carli et al. [30]	Two parallel arms, 2-site, single-blind PREHAB (*n* = 55) REHAB (*n* = 55)	Colorectal ≥ 65 y/o Fried Frailty Index > 1 *n* = 110	PREHAB: 4 weeks REHAB: 4 weeks	Exercise: aerobic and resistance training Nutritional: WPS, Nutritional counselling Psychological: psychology-trained staff, exercises to do at home	6MWD	→
Dunne et al. [42]	Two parallel arms PREHAB (*n* = 20), SC (*n* = 18)	Liver ≥ 18 y/o *n* = 37	4 weeks	Exercise: interval training	VO_2Peak_	↑
Ferreira et al. (a) [32]	Two parallel arm, single-blind PREHAB + REHAB (*n* = 52), REHAB (*n* = 43)	Lung Adult patients *n* = 95	PREHAB: 4 weeks REHAB: 8 weeks	Exercise: aerobic and resistance training Nutritional: WPS, counselling Psychological: psychology-trained staff, exercises to do at home	6MWD	→
Ferreira et al. (b) [31]	Two parallel arms, open-label PREHAB (*n* = 24), SC (*n* = 10)	Lung Adult patients *n* = 34	4 weeks	Exercise: aerobic and resistance training Nutritional: WPS, counselling, EPA, DHA, and Vit. D3 supplementation Psychological: psychology-trained staff, exercises to do at home	6MWD	→
Gillis et al. [33]	Two parallel arm, single-blind PREHAB + REHAB (*n* = 38), REHAB (*n* = 39)	Colorectal Adult patients *n* = 77	PREHAB: 4 weeks REHAB: 8 weeks	Exercise: aerobic and resistance training Nutritional: WPS, counselling Psychological: psychology-trained staff, exercises to do at home	6MWD	↑
Huang et al. [7]	Three parallel arms PREHAB1 (*n* = 30), PREHAB2 (*n* = 30), SC (*n* = 30)	Lung *n* = 90	1 week	Exercise (PREHAB1): aerobic and respiratory training Exercise (PREHAB2): respiratory training	6MWD	↑
Karenovics et al. [43]	Two parallel arms, prospective, open blinded end point PREHAB (*n* = 74), SC (*n* = 77)	Lung ≥ 18 years old *n* = 151	median [IQR] 26 [12, 21–32] days	Exercise: HIIT	VO_2Peak_ 6MWD*	↑
Karlsson et al. [50]	Two parallel arm, feasibility study PREHAB (*n* = 10), SC (*n* = 11)	Colorectal ≥ 70 y/o *n* = 21	median (range) 17 (14–24) days	Exercise: aerobic, resistance, and respiratory training	6MWD	?
Lai et al. (a) [6]	Two parallel arms, single-blind PREHAB (*n* = 30), SC (*n* = 30)	Lung ≥ 70 y/o *n* = 60	1 week	Exercise: aerobic and respiratory training	6MWD	↑
Lai et al. (b) [44]	Two parallel arms, single-blind PREHAB (*n* = 51), SC (*n* = 50)	Lung > 75 y/o *n* = 101	1 week	Exercise: aerobic and respiratory training	6MWD	↑
Liu et al. [34]	Two parallel arms, single-blind PREHAB (*n* = 37), REHAB (*n* = 36)	Lung < 70 y/o *n* = 73	2 weeks	Exercise: aerobic, resistance, and respiratory training Nutritional: WPS, counselling Psychological: exercises to do at home	6MWD	↑
Ma et al. [45]	Three parallel arms, pilot, single-centre, single-blind PREHAB1 (*n* = 34), PREHAB2 (*n* = 32), SC (*n* = 35)	Lung ≥ 70 y/o *n* = 101	2 weeks	Exercise (PREHAB1): aerobic and respiratory training Exercise (PREHAB2): respiratory training	6MWD	↑
Minnella et al. [4]	Two parallel arms, pragmatic, single-blind PREHAB (*n* = 26), SC (*n* = 25)	Esophagogastric ≥ 70 y/o *n* = 51	mean (SD) 42 (43.92) days	Exercise: aerobic and resistance training Nutritional: WPS, counselling	6MWD	↑
Molenaar et al. [35]	Two parallel arms, open-label, international, multicentre, single-blind PREHAB (*n* = 123), SC (*n* = 128)	Colorectal Adult patients *n* = 251	4 weeks	Exercise: resistance and HIIT Nutritional: WPS, counselling, Vit. D and multinutrient supplementation Psychological: psychology-trained staff, exercises to do at home, psychologist referral is required	6MWD	↑
Morano et al. [46]	Two parallel arms, single-blind PREHAB (*n* = 12), SC (*n* = 12)	Lung Patients with previous respiratory disease and impaired respiratory function spirometry *n* = 24	4 weeks	Exercise: aerobic, PNF, and respiratory training	6MWD	↑
Moug et al. [47]	Two parallel arms, feasibility study PREHAB (*n* = 24), SC (*n* = 24)	Colorectal (rectal) > 18 y/o *n* = 48	median [IQR] 14 [13–17] weeks	Exercise: aerobic training	6MWD	?
Santa Mina et al. [36]	Two parallel arm, PREHAB (*n* = 44), SC (*n* = 42)	Prostate 40–80 y/o *n* = 86	mean (SD) 45 (27) days	Exercise: aerobic, resistance, and pelvic floor training	6MWD	↑
Sebio Garcia et al. [37]	Two parallel arms, single-blind PREHAB (*n* = 10), SC (*n* = 12)	Lung ≥ 18 y/o FEV1 ≤ 80% and/or BMI ≥ 30 and/or age ≥ 75 years and/or two or more co-morbidities identified in the Colinet Comorbidity Score *n* = 22	mean (SD) 54.5 (15.4) days	Exercise: aerobic, resistance, and respiratory training	6MWD	→
Stefanelli et al. [5]	Two parallel arms PREHAB (*n* = 20), SC (*n* = 20)	Lung *n* = 40	3 weeks	Exercise: upper and lower body aerobic and respiratory training	VO_2Peak_	↑
Steffens et al. [38]	Two parallel arms, pilot, single-centre, single-blind PREHAB (*n* = 11), SC (*n* = 11)	Abdominal 18–80 y/o *n* = 22	2–6 weeks	Exercise: aerobic and resistance training	6MWD	→
Waller et al. [39]	Two parallel arms, pilot, single-centre PREHAB (*n* = 11), SC (*n* = 11)	Abdominal ≥ 18 y/o *n* = 22	mean (SD) 30.5 (19.8) days	Exercise: aerobic and resistance training Nutritional: Educational material, Fitbit used to log food and protein Psychological: Mindful session via smartphone app	6MWD	↑

*6MWD*, 6-min walk distance; *DHA*, docosahexaenoic acid; *EPA*, eicosapentaenoic acid; *HIIT*, high-intensity interval training; *IQR*, interquartile range; *ONS*, oral nutritional supplement, *PNF*, proprioceptive neuromuscular facilitation; *PREHAB*, prehabilitation; *REHAB*, rehabilitation; *SC*, standard care; *SD*, standard deviation; *Vit*., vitamin; *WPS*, whey protein supplementation; ↑, beneficial effects from Prehab; → , no beneficial effects from Prehab; ?, no conclusion reported

All the studies included patients awaiting surgery for a total of 1994 patients (Fig. 1). Twenty-five studies included one PREHAB group, and another two studies included two PREHAB groups. Group sizes ranged from 10 to 128 patients. In the PREHAB group, group sizes ranged from 10 to 132 patients, with a total of 1040 patients, and in the SC group, groups sizes ranged from 10 to 128, with a total of 954 patients. After selecting patients diagnosed with cancer, and with data available at baseline and at least one more time point (e.g., prior to surgery), the total number of patients was reduced to 1889. In terms of the treatment undergone by patients, twelve studies (44%) included patients undergoing surgery only, while fourteen (52%) included patients undergoing neoadjuvant chemotherapy prior to surgery. Excluding one study (4%), which enrolled patients awaiting prostatectomy, the rest of the studies (96%) included both female and male patients. Studies reported on patients with lung cancer (*n* = 11; 41%), colorectal cancer (*n* = 5; 19%), abdominal cancer (*n* = 3; 11%), esophagogastric cancer (*n* = 2; 7%), liver cancer (*n* = 1; 4%), prostate cancer (*n* = 1; 4%), bladder cancer (*n* = 1; 4%), urological cancer (*n* = 1; 4%), and gastrointestinal cancer (*n* = 1; 4%). Most of the studies compared PREHAB interventions with SC (*n* = 23; 85%), followed by studies comparing a PREHAB and rehabilitation intervention with only rehabilitation (*n* = 3; 11%), and studies comparing a PREHAB with rehabilitation (*n* = 1; 4%). Due to the nature of the non-PREHAB groups, the latter four studies were only included in the meta-analysis comparing preoperative changes in 6MWD. In respect of the length of PREHAB interventions, six (21%) lasted from 1 to 2 weeks, thirteen (45%) lasted from 3 to 4 weeks, and ten (34%) lasted more than 4 weeks. There was an almost equal distribution of studies implementing unimodal (*n* = 14; 52%) and multimodal (*n* = 13; 48%) PREHAB interventions. The exercise component was included in all the studies (100%), nutrition in ten (37%), and psychological support in nine studies (33%). Most of the studies assessed functional capacity at least twice preoperatively (*n* = 26; 96%) and only one (4%) did not assessed functional capacity right before surgery. When adherence or compliance where reported (*n* = 21; 78%), their definition differed amongst studies. Therefore, we could not objectively summarise such outcomes. The 6MWD walk distance was used to measured functional capacity in twenty-two studies (81%) while VO_2Peak_ was used in seven (26%). To summarise the effects of PREHAB on functional capacity, most of the studies (*n* = 17; 63%) reported beneficial effects of PREHAB over SC, followed by studies reporting no differences (*n* = 7; 26%), and studies where data were provided with no reported conclusion (*n* = 3; 11%).

### Changes on the analysed variables

#### Preoperative changes in 6MWD

**Fig. 4:**
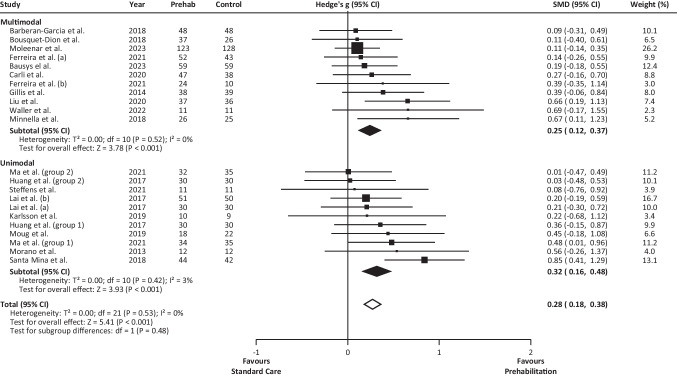
6MWD distance Forest plot. Results of a random-effects meta-analysis showed the effect size (g) with 95% confidence interval. The black diamonds represent the subgroups (unimodal or multimodal) standardised mean differences and the white diamond the pooled (overall) standardised mean differences. 6MWD, 6-min walk distance; CI, confidence interval

From the 21 studies analysing 6MWD, the mean effect size of PREHAB vs. SC was found to be small (*g* = 0.273, 95% CI 0.174 to 0.371) (Fig. 4). The sensitivity analysis showed that none of the studies contributed disproportionately to the results of the meta-analysis. We did not identify any outlier amongst the analysed studies. Inspection of funnel plots revealed more studies to the right of the mean (Online Resource 1). Applying the “trim and fill” procedure, 6 studies were added to the left of the mean, which resulted in an adjusted mean effect size of 0.183 (95% CI 0.064 to 0.301), still showing a positive effect size.

The subgroup analysis revealed a small mean effect size for both unimodal (*g* = 0.318, 95% CI 0.159 to 0.476) and multimodal (*g* = 0.244, 95% CI 0.118 to 0.370) PREHAB interventions (Fig. 4). The analysis also revealed no statistically significant differences between unimodal and multimodal interventions (Q(1) = 0.505, *p* = 0.477).

#### Preoperative changes in VO_2Peak_

**Fig. 5:**
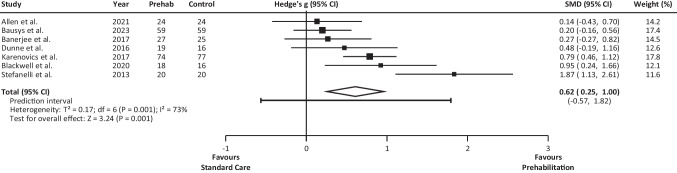
VO_2Peak_ Forest plot. Results of a random-effects meta-analysis showed the effect size (g) with 95% confidence interval. The white diamond represents the pooled (overall) standardised mean differences. CI, confidence interval; VO_2Peak_, peak oxygen consumption

From the 7 studies analysing VO_2Peak_, the mean effect size of PREHAB vs. SC was found to be medium (*g* = 0.615, 95% CI 0.243 to 0.987) (Fig. 5). The prediction interval was calculated from -0.562 to 1.793 (95% CI). The sensitivity analysis showed that none of the studies contributed disproportionately to the results of the meta-analysis. No outliers were identified amongst the analysed studies. Inspection of funnel plots revealed one study more to the left of the mean when compared with the number of studies to the right of the mean (Online Resource 2). However, when applying the “trim and fill” procedure, no studies were added to either side of the mean. Due to the small number of eligible studies, we did not perform any subgroup analysis for this variable.

#### Postoperative changes in 6MWD and VO_2Peak_

Since only three eligible studies assessed 6MWD on discharge [38, 45, 50], and three assessed it after discharge [4, 17, 34], no meta-analysis was conducted. Moreover, only two eligible studies assessed change in VO_2Peak_ during the postoperative period [5, 43]. Therefore, no meta-analysis was conducted for this variable either.

## Discussion

Compared to preoperative SC, PREHAB led to small preoperative improvements in functional capacity as estimated by the 6MWD test. This effect was shown whether the intervention consisted of multimodal PREHAB (i.e., exercise and other components) or unimodal exercise-based PREHAB. The mean effect of PREHAB on VO_2Peak_ was also positive. However, the prediction interval ranged from a medium negative to a very large positive effect size (Fig. 5). Based on the currently available data, we cannot assert that PREHAB always improves VO_2Peak_. This uncertainty derives from the low number of eligible studies, their different effect sizes, and the heterogeneity displayed by their PREHAB programmes. In fact, only two of the studies implemented multimodal PREHAB, including exercise, nutrition, and psychological support [40, 49]. The other five studies featured unimodal exercise-based PREHAB and, despite implementing aerobic exercise, the training protocols varied widely amongst studies [5, 28, 41–43]. Interestingly, the studies by Allen et al. [40] and Bausys et al. [49] displayed the least favourable effect size of PREHAB over SC (Fig. 5) despite implementing longer-lasting multimodal PREHAB programmes (ranging from 8 to 15 weeks) (Table 1), compared to the shorter unimodal exercise-based PREHAB programmes (ranging from 3 to 4 weeks) implemented in the rest of the studies.

We considered the possibility of neoadjuvant chemotherapy impacting the effect of PREHAB. Coincidentally, we found that the three studies [5, 28, 43] with the greatest effect size and both 95% CI favouring PREHAB had a low percentage of patients undergoing neoadjuvant chemotherapy, ranging from 0 to 12% (Fig. 5). In contrast, the proportion of patients undergoing neoadjuvant chemotherapy was higher in the other four studies [40–42, 49], ranging from 33 to 100%. The exposure to chemotherapy treatment could hinder the ability of patients to exercise, and consequently to develop the physiological adaptations expected from PREHAB, hence, explaining the different effect size observed between studies. Notwithstanding the potential interference between chemotherapy and PREHAB-induced improvements on functional capacity, exercise-based interventions provide a beneficial immune effect without adding to the common side effects experienced due to chemotherapy treatment [52]. Furthermore, exercise-based interventions could aid to improve the common cancer-related health outcomes such as fatigue, anxiety, depression symptoms, and health-related quality of life [53]. Additionally, the inclusion of resistance training would induce beneficial effects against muscle atrophy, a prevalent side effect of chemotherapy [54]. However, to be beneficial, interventions should be tailored to the individual characteristics of each patient [52] and the training variables should be manipulated to provide the expected physiological adaptations [55].

From a practical perspective, even though no statistical heterogeneity was determined in the analysis of 6MWD, it is worth noticing some methodological differences between the included studies. Exercise-based programmes can vary widely even when implemented as a unimodal intervention. The almost endless number of combinations of exercise modalities (e.g., resistance, aerobic, respiratory training) and configurations of training variables (e.g., volume, intensity, inter-set rest periods, and frequency) contributes to a great diversity of approaches. Therefore, implementing multimodal programmes implies adding more complexity, and potentially increasing the methodological divergence between studies. Indeed, despite all the included studies implemented an exercise component, the difference amongst programmes was evident, with some of them containing exclusively breathing exercises [7, 45] and others combining aerobic, resistance, and breathing exercise [34, 37, 38, 40, 49, 50]. When nutrition was included in the intervention, the most common strategy was individualised nutritional counselling with an emphasis on appropriate protein intake by providing protein supplements [4, 29–35, 49], such as whey concentrates or isolates [4, 29–35]. Other supplements, such as omega-3 fatty acids, vitamin D3, and multi-ingredient formulations [30–32, 35, 49], were also used. Anxiety management strategies were the main focus within the psychological support component [29–35, 39, 40, 47–49]. Due to the scarcity of eligible studies integrating nutrition into multimodal PREHAB, it was difficult to ascertain the effect of nutrition on the functional capacity of surgical oncology patients and whether it can provide similar benefits to exercise-based interventions.

The present meta-analysis is unique due to the chosen eligibility criteria, the rigour of selecting the included studies, and the fact that we have analysed changes in functional capacity (e.g., from baseline to before surgery) instead of analysing static values (e.g., before surgery, post-intervention). Our review included data from patients who had a confirmed diagnosis of cancer with measurements at baseline and at least another time point (e.g., prior to surgery) [48, 50]. Despite methodological differences, our results agree with previous systematic reviews with meta-analyses of RCT supporting the notion that PREHAB can improve patient’s functional capacity prior to oncological resections [10, 11, 13–19].

For instance, meta-analyses of RCTs studying the effect of PREHAB in patients with lung cancer have reported enhancements in their functional capacity [10, 11, 13, 16, 18]. Pu et al. [11] meta-analysed four studies and reported improvements on 6MWD after patients completed preoperative breathing exercises. Moreover, Gravier et al. [13] analysed the effects of aerobically demanding exercise PREHAB programmes finding worthwhile positive effects on 6MWD when comparing PREHAB to SC. Simultaneously, these authors summarised data on VO_2Peak_ and found PREHAB to be beneficial. Furthermore, Cavalheri et al. [10] reported favourable effects of exercise-based PREHAB vs. SC on functional capacity by means of the 6MWD. Along those lines, Li et al. [16] and Rosero et al. [18] found significant differences favouring exercise-based PREHAB interventions when compared to SC as estimated by 6MWD and VO_2Peak_. Although we included any cancer speciality, the outcomes of our meta-analysis align with the results of the aforementioned reviews, supporting the beneficial effects of PREHAB on functional capacity in patients with lung cancer.

On the other hand, van Gestel et al. [14] reported positive effects of home-based multimodal PREHAB on the functional capacity of patients after oncological resections from any cancer speciality. Compared to control groups receiving SC, PREHAB improved 6MWD pre-surgery and at 8 weeks post-operatively. Similarly, Smyth et al. [20] performed a meta-analysis comparing the effects of high-intensity interval training PREHAB vs. SC in oncological resections. The authors observed non-significant differences between PREHAB and SC on VO_2Peak_ values. Due to our eligibility criteria, we could not analyse the postoperative effects of PREHAB on functional capacity. However, the preoperative outcome analysis reported by van Gestel et al. [14] and Smyth et al. [20] reinforce our findings that the analysis of the included RCT would not unequivocally support that PREHAB improves VO_2Peak_. Nonetheless, when analysing the impact of PREHAB on 6MWD, we found more robust and consistent evidence that PREHAB can improve preoperative functional capacity vs. SC.

The effects of PREHAB programmes on exercise capacity of surgical gastrointestinal cancer patients have also been studied by Lau et al. [15]. The authors included studies featuring unimodal or multimodal PREHAB programmes. Their preoperative analysis revealed improvements elicited by PREHAB on 6MWD. These findings remained true for their postoperative analysis, at 4–8 weeks after surgery. Our findings and those by Lau et al. [15] support the notion that both unimodal and multimodal PREHAB programmes can improve functional capacity in patients with cancer.

In relation to abdominal oncological resections, Waterland et al. [19] investigated the effect of PREHAB on functional capacity of patients undergoing such procedures. By analysing three studies, the authors observed no significant improvement in VO_2Peak_. Furthermore, a meta-analysis on 6MWD preoperative changes showed an improvement on 6MWD elicited by PREHAB when compared to SC. Both findings are strengthened by the outcomes of our analysis. Waterland et al. [19] also performed a subgroup analysis for the 6MWD analysis and showed benefits of PREHAB to remain significant for multimodal programmes and, contrary to our findings, non-significant for unimodal programmes. We believe this difference could be due to (a) a more restrictive inclusion criteria (i.e., abdominal oncological resections), (b) the limited number of studies analysed by the authors (two) in contrast with those analysed in our meta-analysis (9 studies, with 11 groups in total), and (c) the inclusion of one study that accepted both patients with cancer and patients with benign disease.

Our results also agree with previous reviews suggesting clinical benefits of PREHAB vs. the SC in patients with colorectal cancer. In this respect, Molenaar et al. [17] reported significant and clinically favourable effects on 6MWD of PREHAB vs. the SC in patients waiting for colorectal resection. In two further analyses, the authors summarised data on 6MWD at 4 and 8 weeks postoperatively and found non-statistically significant benefits of PREHAB over SC. Additionally, our results agree with the meta-analysis by Falz et al. [12] supporting the effects of PREHAB to significantly improve 6MWD in patient awaiting colorectal cancer resections.

## Limitations and recommendation for future studies

Several aspects of this review must be considered when attempting to draw evidence-based inferences. The broad inclusion criteria implemented in this review allowed for the analysis of studies including patients from various cancer specialities such as lung, colorectal, abdominal, esophagogastric, liver, urological, and gastrointestinal. Therefore, while the findings of this review may be applied to these types of cancers, caution is granted when considering other cancer specialities (e.g., brain, breast). Future RCT should investigate the effect of PREHAB on functional capacity in patients diagnosed with other cancers not yet explored.

Most of the studies included in our meta-analysis assessed 6MWD as a measurement of functional capacity and only a few used VO_2Peak_. Although the 6MWD analysis revealed that PREHAB provides a small benefit on functional capacity, the outcomes of the VO_2Peak_ analysis did not show such certainty. We suggest that a greater number of studies assessing VO_2Peak_ could increase statistical power and help to gain insight into the effects of PREHAB on oxygen uptake.

Although this review shows how PREHAB improves preoperative functional capacity, we have not analysed whether this improvement also represents better surgical outcomes. As this is important from a clinical perspective, further research should explore the correlation between preoperative functional capacity changes and surgical outcomes.

In this review, 6MWD and VO_2Peak_ were selected due to being well-recognised measurements of functional capacity. However, the authors recognised the presence of other tests that could also be relevant to assess functional capacity (e.g., timed up-and-go test, 60-s sit-to-stand test). We did not analyse the independent effect that different components could have on functional capacity. As such, we could not ascertain whether nutrition- or psychological-based unimodal PREHAB could be comparable to exercise-based unimodal PREHAB programmes. In this regard, nutrition, and particularly protein intake, could play a major role in retaining lean body mass, which could positively impact functional capacity. Future studies implementing non-exercise-based unimodal PREHAB should assess functional capacity to shed light onto this hypothesis.

## Conclusions

Our results show that prehabilitation, whether implemented as unimodal or multimodal format, elicits small preoperative functional capacity improvements in patients awaiting oncological resections. These benefits seem to extend to various tumour groups. Clinicians are encouraged to integrate prehabilitation programmes including at least an exercise component as part of their patients’ cancer care pathway.

## Supplementary Information

Below is the link to the electronic supplementary material.

Supplementary file1 (DOCX 19 KB)

## Data Availability

No datasets were generated or analysed during the current study.
