# Supplementary material for: Effect of prehabilitation programmes on functional capacity in patients awaiting oncological resections: a systematic review and meta-analysis of randomised controlled trials
Source: Support Care Cancer. 2024 Sep 17;32(10):667. doi: 10.1007/s00520-024-08875-8 (PMC11408567; doi:10.1007/s00520-024-08875-8)
Supplement: Supplementary file 1 — Supplementary file1 (DOCX 19 KB) [file 520_2024_8875_MOESM1_ESM.docx]

**Effect of Prehabilitation Programmes on Exercise Capacity in Patients Awaiting Oncological Resections: A Systematic Review and Meta-analysis of Randomised Controlled Trials**

Roberto Laza-Cagigas, Eneko Larumbe-Zabala, Marcos Seijo, Tara Rampal and Fernando Naclerio

Supportive Care in Cancer

**Corresponding author**

Fernando Naclerio

Institute for Lifecourse Development, Centre for Exercise Activity and Rehabilitation, School of Human Science. University of Greenwich, Eltham SE9 2TB, United Kingdom.

Email: [f.j.naclerio@greenwich.ac.uk](mailto:f.j.naclerio@greenwich.ac.uk)

**Online Resource 1** 6MWD funnel plot

**Online Resource 2** VO_2Peak_ funnel plot
